# Mechanical Analysis of Single Myocyte Contraction in a 3-D Elastic Matrix

**DOI:** 10.1371/journal.pone.0075492

**Published:** 2013-10-03

**Authors:** John Shaw, Leighton Izu, Ye Chen-Izu

**Affiliations:** 1 Department of Aerospace Engineering, University of Michigan, Ann Arbor, Michigan, United States of America; 2 Department of Pharmacology, University of California Davis, Davis, California, United States of America; 3 Departments of Biomedical Engineering, Pharmacology, Medicine/Cardiology, University of California Davis, Davis, California, United States of America; University of Debrecen, Hungary

## Abstract

**Background:**

Cardiac myocytes experience mechanical stress during each heartbeat. Excessive mechanical stresses under pathological conditions cause functional and structural remodeling that lead to heart diseases, yet the precise mechanisms are still incompletely understood. To study the cellular and molecular level mechanotransduction mechanisms, we developed a new ‘cell-in-gel’ experimental system to exert multiaxial (3-D) stresses on a single myocyte during active contraction.

**Methods:**

Isolated myocytes are embedded in an elastic hydrogel to simulate the mechanical environment in myocardium (afterload). When electrically stimulated, the in-gel myocyte contracts while the matrix resists shortening and broadening of the cell, exerting normal and shear stresses on the cell. Here we provide a mechanical analysis, based on the Eshelby inclusion problem, of the 3-D strain and stress *inside* and *outside* the single myocyte during contraction in an elastic matrix.

**Results:**

(1) The fractional shortening of the myocyte depends on the cell’s geometric dimensions and the relative stiffness of the cell to the gel. A slender or softer cell has less fractional shortening. A myocyte of typical dimensions embedded in a gel of similar elastic stiffness can contract only 20% of its load-free value. (2) The longitudinal stress inside the cell is about 15 times the transverse stress level. (3) The traction on the cell surface is highly non-uniform, with a maximum near its ends, showing ‘hot spots’ at the location of intercalated disks. (4) The mechanical energy expenditure of the myocyte increases with the matrix stiffness in a monotonic and nonlinear manner.

**Conclusion:**

Our mechanical analyses provide analytic solutions that readily lend themselves to parametric studies. The resulting 3-D mapping of the strain and stress states serve to analyze and interpret ongoing cell-in-gel experiments, and the mathematical model provides an essential tool to decipher and quantify mechanotransduction mechanisms in cardiac myocytes.

## Introduction

Cardiac muscle contraction generates mechanical force to pump blood, so the muscle cell experiences mechanical stress during each heartbeat. Excessive mechanical stress associated with pathological conditions, such as hypertension, volume overload, infarction, and asynchronous contraction, can result in cardiac remodeling and heart disease development [1]. Although the link between mechanical stress and cardiac remodeling is well known, the cellular and molecular mechanisms that transduce mechanical stress in myocytes remain incompletely understood.

Previously, studies on cardiac excitation-contraction mechanisms were mostly conducted using myocytes under *load-free* conditions. Investigation of the mechanotransduction mechanisms has been hindered by lack of techniques to control the mechanical load at single cell level, especially in the case of live adult cardiac myocytes. Pioneering studies developed techniques to apply longitudinal stretch to the single cell. Kohl and colleagues [2] used carbon fibers attached to the cell’s opposite ends to control force and strain, and explored the stress-strain relationship under various preloads. Petroff et al. [3] and Prosser et al. [4] found that stretching myocytes caused spontaneous Ca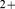 sparks and waves. These and other studies provided exciting new research avenues to understand and quantify the significant impact of the preload on the myocytes.

Precisely how mechanical stress in 3-dimensional (3-D) tissue affects the myocyte is still unknown. Under physiological conditions, the myocyte undergoes contraction and relaxation in synchrony with surrounding cells to pump blood against the pressure imposed by circulatory resistance (the afterload). In addition, under pathological conditions, such as infarction and asynchronous contraction (i.e. arrhythmias, fibrillation), a myocyte may contract asynchronously against its neighbors and experience more severe and complex multiaxial mechanical stresses imposed by the surrounding myocardium.

**Figure 1:**
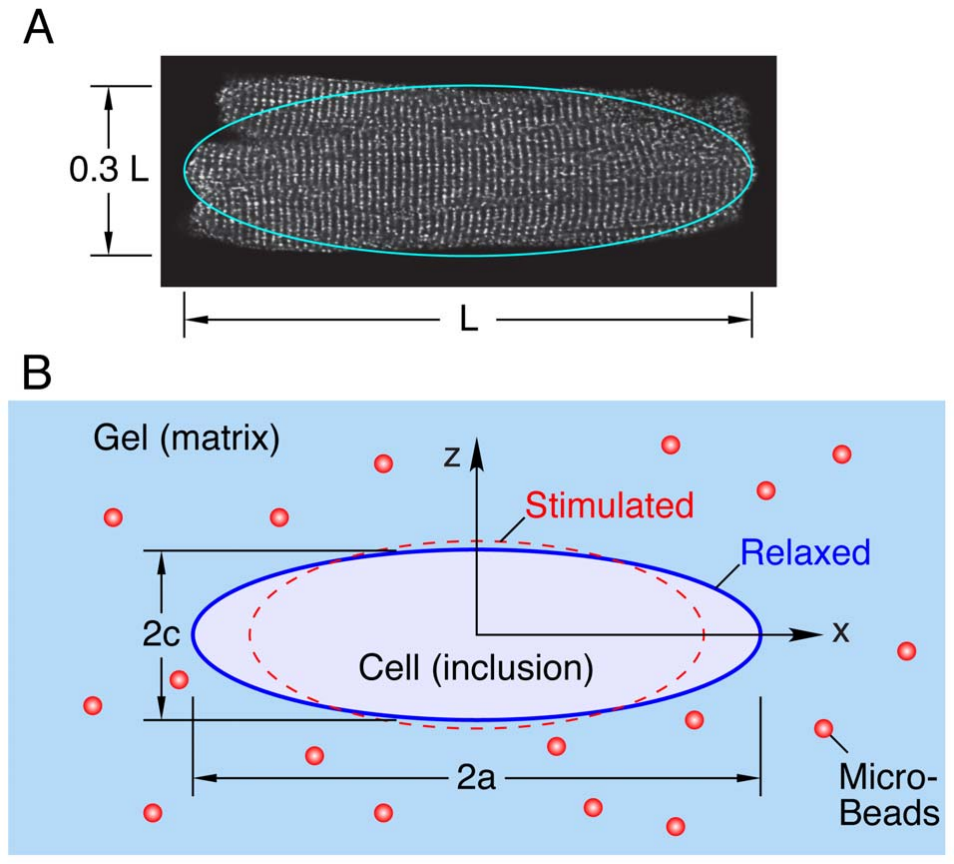
Boundary value problem analyzed. (A) confocal micrograph of cardiomyocyte, (B) schematic of cell-in-gel experiment (contracted configuration of cell exaggerated).

To investigate how multiaxial mechanical stress may affect the myocytes, we developed a cell-in-gel system by embedding live myocytes in a 3-D elastic hydrogel matrix. The gel is made of poly vinyl alcohol (PVA) and tetravalent boronate-PEG crosslinker [5]. The cell surface is adhered to the gel by crosslinking the hydroxyl groups in the extracellular matrix. When electrically stimulated, the in-gel myocyte contracts against the elastic matrix, and the matrix resists shortening and broadening of the cell during contraction, exerting multiaxial mechanical stress on the cell. Along with the myocyte, micro-beads are embedded in the gel, and confocal imaging can be used to measure myocyte dimensional changes and micro-bead displacements as the cell contracts (see Fig. 1). While it is extremely difficult to exactly simulate the various in vivo conditions, the impact of mechanical stresses on single myocytes can be studied using the cell-in-gel system in a controlled way. The Young’s modulus of the gel is adjustable by the ratio of PVA and crosslinker concentrations, so the gel stiffness can be changed/tuned to approximate a range of afterload conditions in myocardium.

In this article, we provide a 3-D mechanical analysis of the single myocyte contraction in-gel. The purpose is to provide a quantitative tool to guide, analyze and interpret ongoing cell-in-gel experiments, which are ultimately aimed at deciphering the cellular and molecular mechanisms of mechano-transduction in the beating heart. Those experiments are generating a large body of data, and the results will be reported elsewhere. Here, we focus on the mathematical model that provides a foundational analysis tool to quantify and map the 3-D mechanical fields *inside* and *outside* the myocyte when it contracts within an elastic matrix. The model provides the interior (cell) and exterior (matrix) displacement fields that can be directly compared and calibrated to experimental imaging measurements in terms of fractional shortening of the myocyte and micro-bead displacements in the matrix. With knowledge of the gel stiffness, the model can be used to calculate the multiaxial stress state inside the cell, the traction distribution on the cell surface (adjacent to chemotransduction sensors), the stress and strain distributions in the gel (if of interest), and the elastic energy expended by the cell. The remainder of this article provides the theoretical development of the model, a parameter study to highlight general trends and gain insights for cell-in-gel experiments, and a discussion of implications and extensions.

## Methods

### Eshelby Inclusion Theory

Of interest is the boundary value problem of a single beating cardiomyocyte embedded in an elastic hydrogel of infinite extent (see Fig. 1). The theory follows closely the classic work of J.D. Eshelby [6], [7] as detailed in [8]. The elasticity problems originally addressed by Eshelby are based on the *inclusion problem* where a subregion (the inclusion) of an infinite, homogeneous, linear elastic solid undergoes a spontaneous change of shape (transformation strain). Since the inclusion is constrained by the surrounding elastic matrix, a residual state of stress is created inside and outside the inclusion and the inclusion is restrained from achieving its new, stress-free, configuration. Likewise, we envision the cardiac cell attempting to contract, but its deformation is constrained by the surrounding gel and only a partial contraction strain is achieved. Eshelby went on to address the inhomogeneous inclusion problem, where the elastic moduli of the subregion are different from the surrounding matrix, by formulating the equivalent inclusion problem. The usual purpose is to calculate the homogenized elastic properties of a composite material [9]–[11], but that is not our aim here. Rather, we seek the detailed stress and strain fields arising directly from the cell’s contraction.

Our key assumptions are:

1. The cell is treated as a homogeneous ellipsoidal inclusion.
2. The cell and gel behave as isotropic linearly elastic solids.
3. Displacements, rotations, and strains are small, allowing linearized kinematics.
4. Cell contraction is isovolumic (isochoric) and occurs uniformly throughout its volume.
5. The cell membrane is adhered to the gel.
6. Remote boundaries of the gel are load-free.
7. The analysis applies to a single myocyte in gel, without mechanical interactions with other cells.

Most of these assumptions, except perhaps the first two, are reasonable considering the cell-in-gel experiments we have mind. We recognize that typical cardiomyocytes are not ellipsoids (often irregular brick-like shapes) and the cell and/or gel may not be linearly elastic (likely somewhat nonlinear and viscoelastic). For now, however, we are content to accept these assumptions, since they greatly simplify the analysis and give a useful first-order analysis that can be extended later if needed.

A fixed Cartesian frame is used with orthonormal base vectors 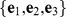 aligned to the principal axes of the ellipsoidal inclusion (cell), and the origin is taken at its centroid. The current procedure for the cell-in-gel experiment starts by embedding the myocyte in a resting state in the PVA solution, and then adding the crosslinker to solidify the gel and adhere it to the cell. Thus, we take the ‘slack’ myocyte adhered to the stress-free gel as our reference configuration. Linearized kinematics are assumed, so field quantities are all functions of referential coordinates 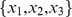. Using indicial notation (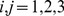), the components of displacement, strain, and stress are respectively 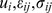, and the summation convention is employed for index pairs, such as 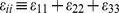. Scalar quantities are written in normal type, while vector and tensor variables are distinguished by bold face. For example, the full notation for the position vector is 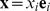. Since field quantities will be understood by their context and the base vectors are fixed, for simplicity the explicit argument 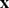 will be suppressed and we can work solely with components of tensorial quantities. Differentiation with respect to spatial coordinates 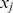 is denoted by the comma subscript, such as the components of the displacement gradient tensor 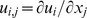. (See Table 1 for nomenclature used throughout.).

**Table 1:** Nomenclature.

*x_i_*	spatial coordinates (*x*_1_, *x*_2_, *x*_3_) = (*x*,*y*,*z*)
*u_i_*	displacement vector components (*u*_1_, *u*_2_, *u*_3_) = (*u*,*v*,*w*)
*ε_ij_*	strain tensor components
*e_ij_*	elastic strain tensor components
*β_ij_*	transformation strain tensor components
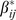	eigenstrain tensor components
*σ_ij_*	stress tensor components
*δ_ij_*	Kronecker delta components
*δ_ijkl_*	4th-order identity tensor components
*t_i_*	traction vector components
*n_i_*	unit normal vector components
*s_i_*	unit tangent vector components
*V* ^I^, *V* ^M^	inclusion, matrix subregions
*a_i_*	ellipsoid principal axes (*a*_1_, *a*_2_, *a*_3_) = (*a*,*b*,*c*)
*V*_e_	ellipsoid volume
*S*	inclusion-matrix interface surface
*μ*,*κ*	shear modulus, bulk modulus
*C_ijkl_*	elasticity modulus tensor components
*E* ,*v*	Young’s modulus, Poisson’s ratio
*φ*, *ψ*	potential functions
*B_ijk_*, *D_ijkl_*	displacement, strain tensor operators
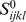	Eshelby tensor components
*λ*	Eshelby parameter
*I*, *I_i_*, *I_ij_*	Eshelby integrals
*F*, *E*	elliptic integrals of 1st & 2nd kind
*θ*, *k*	elliptic integral arguments
*M_ij_*, *N_ij_*	defined intermediate integrals
*Q*, *ω*	defined constants
*α_k_*, *A*, *h*, *G*, *J*, Λ, Γ, *P*	defined intermediate functions
*η*	matrix/inclusion modulus ratio
*U*	mechanical strain energy

### The Homogeneous Inclusion Problem

The boundary value problem of interest is a solid body of infinite extent (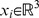), which includes an inclusion (cell) sub-volume denoted 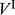 bounded by a closed surface 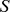. The remainder exterior volume, occupied by the matrix (gel), is 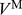. Initially, the stress and strain fields in both domains are zero when the cell is relaxed. Under load-free stimulation, the cell would contract by a uniform inelastic strain 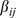, but due to the presence of the gel the cell achieves a constrained strain of 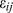, which is what we seek. In the absence of body forces, the equilibrium field equations for the components of the stress tensor (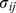) and traction vector (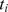) are
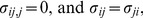
(1a)

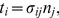
(1b)where 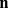 is the unit normal (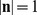) to a surface. Equations (1a) and (1b) represent equilibrium of volumetric and surface elements, respectively, both of which apply wherever the fields are sufficiently smooth and differentiable. The matrix is assumed to be unloaded at remote boundaries, so 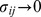 as 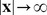.

Since the inclusion and matrix are bonded at surface 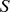, the displacements 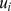 are continuous, yet some of their derivatives may be discontinuous, so the following ‘jump’ conditions are enforced along 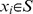


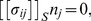
(2a)

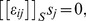
(2b)where here 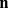 is the unit outward normal to 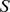, and 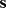 is any orthogonal tangent vector in 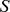 (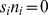). The notation 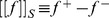 denotes the jump in quantity 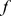 with limiting values 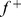 and 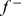 on outer and inners sides of 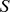, respectively. Equation (2a) enforces equilibrium of surface elements in 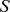, while eq. (2b) enforces continuity of *in-surface* strains stemming from displacement continuity (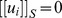).

The infinitesimal strain-displacement relations in linear elasticity theory are
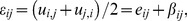
(3)with the strain decomposed into elastic (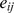) and inelastic (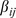) parts, and 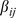 represents the constant, (stress-free) transformation strain in the inclusion (taken to be zero in the matrix). The constitutive equations for stress are 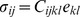, where 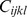 are the 4th-order stiffness tensor components, which if isotropic gives



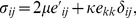
(4a)

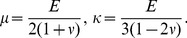
(4b)


Here, the elastic strain is decomposed further into deviatoric (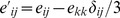) and dilatational (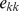) parts, 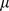 and 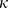 are the respective shear and bulk moduli (also given in terms of Young’s modulus 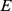 and Poisson’s ratio 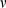), and 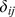 is the Kronecker delta. For now, we are considering the *homogeneous* inclusion problem where the properties 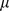 and 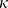 are common to the inclusion and matrix (only 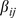 is different), but this will be relaxed later when we consider the *inhomogeneous* inclusion problem.

#### Solution for an isotropic, ellipsoidal inclusion

The general solution found by Eshelby was expressed in terms of the following scalar-valued fields
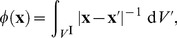
(5a)

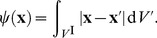
(5b)


The quantities 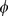 and 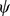 are harmonic and biharmonic potential functions, respectively, that satisfy
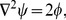
(6a)

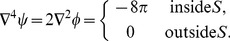
(6b)


The potentials, 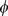 and 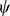, are smooth (analytic) functions at all points except along 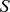 (with unit outward normal components 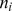), where they suffer discontinuities in the following derivatives
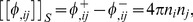
(7a)

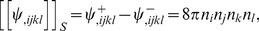
(7b)with superscripts 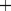 and 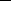 denoting respective quantities evaluated just outside or inside 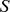.

We consider the case of an ellipsoidal inclusion, which besides being a rather versatile object in analysis, is the only known shape where the strain and stress fields inside the inclusion are uniform [12], [13]. This greatly simplifies the calculation. The boundary of the ellipsoid has principal axes, ordered as 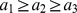, and the domain of 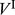 is
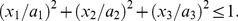
(8)


The volume of the ellipse is 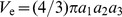.

When isotropic properties are used the displacement and strain fields are
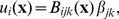
(9a)

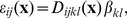
(9b)with



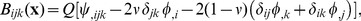
(10a)

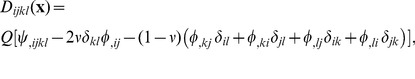
(10b)where 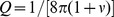. The tensors 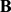 and 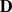 will be expressed in terms of the following integrals



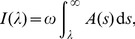
(11a)

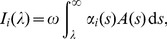
(11b)

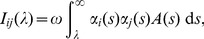
(11c)with 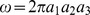, 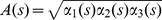 and 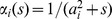. The argument of the integrals (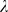) is zero for all points inside the inclusion, and for exterior points is taken as the largest positive root of



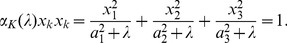
(12)Here, we adopt the modified index convention as used in [8], in which repeated *lower* case indices are summed as usual, but repeated *upper* case indices are not summed. Instead, the upper case indices just take the same value as their lower case counterparts. For example, the equation of the ellipsoid surface can be written in this way as simply 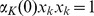. The resulting potentials are
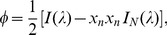
(13a)

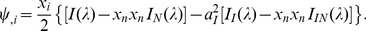
(13b)


To evaluate eq. (10), we need the higher derivatives of 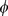 and 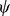. The first derivatives of eq. (11) are
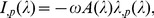
(14a)

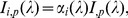
(14b)

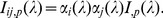
(14c)


From here on the 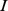-integrals, 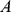 and 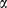
′s are understood to be functions of 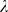, so the argument will be dropped for simplicity. Note that by eq. (12) the derivative of bracketed expressions in eq. (13) reduce to
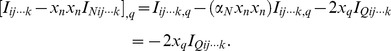
(15)


Using again eq. (12) and taking derivatives of eqs. (13a) and (13b) gives
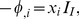
(16a)

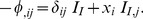
(16b)

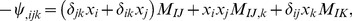
(16c)




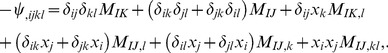
(16d)with the definition 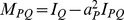. With this, eqs. (10a) and (10b) become



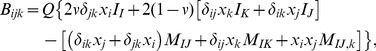
(17a)

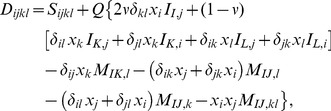
(17b)with



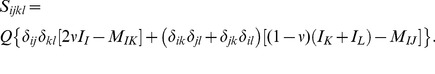
(17c)This result is valid for exterior and interior points, recallingzzzz that the 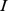-integrals are functions of 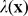 in the matrix, but 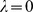 on the boundary 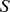 and inside the inclusion. Inside an *ellipsoidal* inclusion all derivatives of the 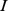
′s and 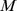
′s vanish, resulting in 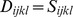 (constant), which is the well-known Eshelby tensor (denoted from here on as 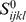).

Following [8], the 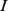-integrals above are expressed in terms of elliptic integrals (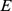 and 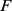) as
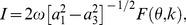
(18a)

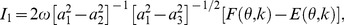
(18b)

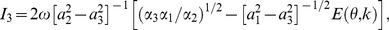
(18c)

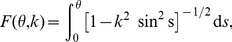
(18d)

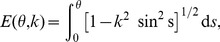
(18e)

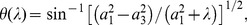
(18f)

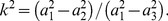
(18g)


Knowing only 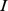, 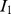 and 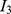 is sufficient, since other 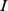-integrals can be found from the relations
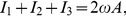
(19a)

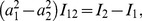
(19b)

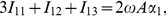
(19c)and the rest from cyclic permutation of indices 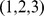. Note that 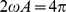 for interior points when 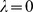.

Computing the required derivatives of 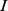
′s in eq. (17) are given below. First, it is convenient to define the following functions
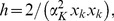
(20a)

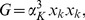
(20b)

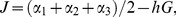
(20c)

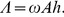
(20d)


Differentiating eq. (12) by 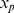, solving for 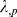, and then differentiating again gives
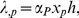
(21a)

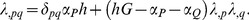
(21b)


From eqs. (14) and (21a), the first derivatives of the 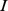-integrals are then
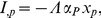
(22a)

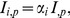
(22b)

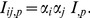
(23c)


From eqs. (21) and (22), and the fact that 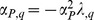 we obtain the second derivatives
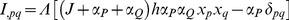
(23a)

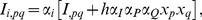
(23b)

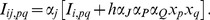
(23c)


Using eqs. (14), (21a), and (22), derivatives of *M*-quantities in eq. (17b) can be written



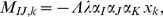
(24a)

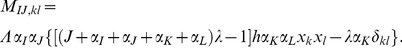
(24b)


Finally, the displacement and strain fields are computed by eqs. (9a) and (9b), and the stresses are found from
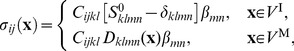
(25)where 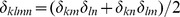 are the components of the 4th-order symmetric identity tensor.

#### The isochoric, incompressible limit

The deformation of biological materials and most soft polymers can reasonably be considered as isochoric (volume preserving, i.e., 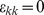 when 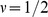) and incompressible continua, since by eq. (4b) the typical bulk modulus 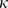 is quite large compared to the shear modulus 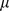. In the limit 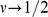 relevant to the cell-in-gel problem, the problem simplifies somewhat. In general the dilatation in the inclusion is 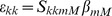, which reduces by eq. (19) to
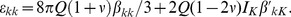
(26)using the deviator of the transformation strain (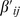). Thus, when the transformation strain is isochoric (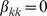) and the material is incompressible (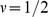), the constrained inclusion strain is also isochoric (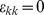) and 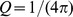. From here on we will assume the following isochoric, cylindrical form for 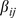


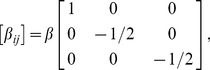
(27)where 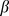 is a negative material constant (simulating cell contraction). The explicit inclusion strains are then



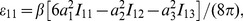
(28a)

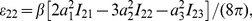
(28b)

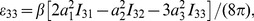
(28c)

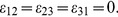
(28d)


Decomposing the inclusion stress (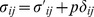) into its deviator (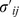) and mean stress (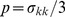), gives
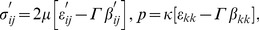
(29a)

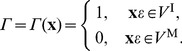
(29b)


The difficulty is that as 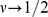, 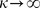 while 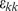 and 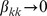, leaving 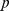 seemingly indeterminate. Returning to the general strain equation, eqs. (16) and (18), the dilation is
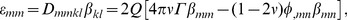
(30a)


The elastic part is.
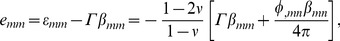
(31)


When eq. (31) is multiplied by 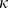 (see eq. (4b)), we get a finite limit due to the canceling factors 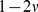,
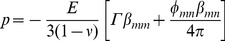
(32)


Specializing to the form of 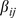 in eq. (27), taking 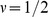 and using eqs. (16b) and (22), the mean hydrostatic stress in the matrix and in the inclusion are
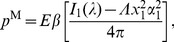
(33a)

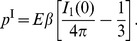
(33b)


### The Inhomogeneous Inclusion

So far, we considered the case where the elastic properties of the inclusion and matrix are the same (aside from 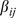). If they are different, similar calculations can be done using the “equivalent inclusion” method [8]. The stresses in the inclusion and matrix are, in general,
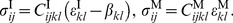
(34)


The approach is to introduce a fictitious transformation strain (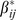), or “eigenstrain”, to simulate the perturbed elastic fields due to the inhomogeniety. This is used to replace the elastic properties of the inclusion with those of the matrix, while preserving the correct stresses in 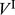


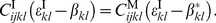
(35)


With appropriate choice of eigenstrains, we just solve the previous homogenous inclusion problem with the new transformation strain (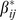), giving the inclusion strains as
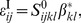
(36)where 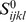 are again the components of the Eshelby tensor. The rest of the solution method is the same as before.

The new task is to determine 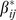, which is algebraic but not trivial. Substituting eq. (36) in eq. (35) gives
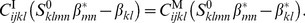
(37)


Rearranging, we get
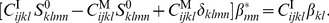
(38)


Equations (38) represent algebraic equations to solve for the six unknown eigenstrains (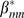). In general the explicit equations are rather messy, so they are not written here. In the isotropic, isochoric (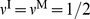) case the equations simplify somewhat. One can show that 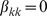 implies 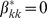. Taking 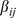 as given in eq. (27) results in
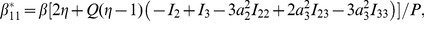
(39a)

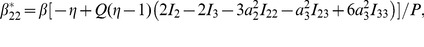
(39b)

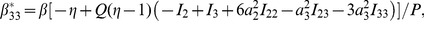
(39c)

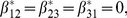
(39d)with the additional definitions



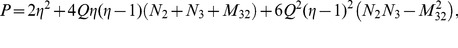
(39e)

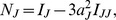
 (39f)and 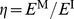 is the ratio of Young’s moduli (or equivalently, shear moduli) of the matrix and inclusion.

#### Mechanical Energy

Once the stress in the inclusion is known, Eshelby also showed that the elastic strain energy (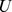) of the entire system (inclusion+matrix) takes a surprisingly simple form, based only on the stress in the inclusion and its transformation strain. The elastic strain energy of the system is 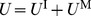, where the respective energies in the inclusion and the matrix are
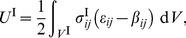
(40a)

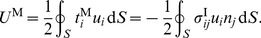
(40b)


In eq. (40b), we started with the traction (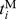) applied to the matrix along 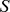. Equilibrium of the surface requires that the traction on the inclusion be 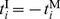, which has the associated stress inside the inclusion 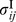 and the outward normal 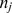. Now Gauss’s theorem is used to convert to a volume integral over the inclusion,
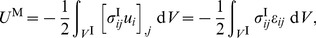
(41)where we used equilibrium eq. (1a) and fact that 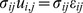 by symmetry of 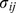. When added to eq. (40a), we are left with only
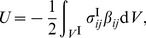
(42)which is a rather convenient result. The entire elastic energy can be calculated from the inclusion stress 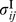 and the stress-free transformation strain 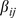. There is no need to calculate the solution outside the inclusion. Furthermore, for the ellipsoidal inclusion the integrand is independent of 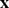, so



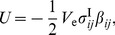
(43a)

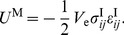
(43b)


Equation (43a) gives the total strain energy in the system, which is potentially useful to determine energy requirements for the cell. Equation (43b) is the strain energy in the matrix, which is just the mechanical work done on the gel by the cell. Both results are valid for the homogeneous and inhomogeneous inclusions, where 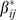 would be used in the calculation of 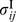 and 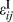, but the actual transformation strain 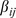 would still multiply the stress in eq. (43a).

## Results and Discussion

With the general analysis complete, explicit calculations are presented below. The fact that an analytical solution is available is fortunate, and this readily facilitates easy parametric studies of the problem. In particular, we are interested in predicting the constrained strain state in the cell, knowing its load-free contraction 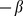 (fractional shortening). If the elastic properties of the cell and gel are known, one can then estimate the average stress state in the cell. Also, since the cell-in-gel experiments include a dispersion of small beads in the gel, their measured displacements during cell contraction can be compared to the predicted displacement field in the gel matrix to further validate the analysis. Cell-in-gel measurements are ongoing and such a comparison will be done elsewhere. For now, we use typical values to get a sense of expected values and trends with respect to relevant parameters in myocytes.

From here on it is convenient to take the principal axes of the cell as 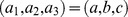, spatial coordinates as 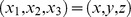, and displacement components as 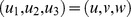. The cell and gel both undergo isochoric (constant volume) deformations, so we take 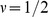 and assume the transformation strain in the cell is of the form given in eq. (27). All results below were calculated in Mathematica v.8.

Although cell to cell variability exists, a typical healthy, ventricular myocyte is about 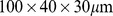, and in our preliminary experiments, isolated myocytes contract by about 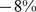 to 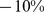 in an un-crosslinked fluid medium (load-free). Thus, we take as a nominal cardiomyocyte 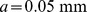, 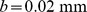, 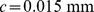, and 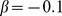.

### Homogeneous Inclusion Analysis

#### Strain knockdown

Assuming the shear moduli of the cell and the gel are the same (so-called homogeneous inclusion) and using eq. (28) with the geometry of our nominal cardiomyocyte, the calculated strain state in the constrained inclusion is
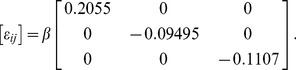
(44)


**Figure 2:**
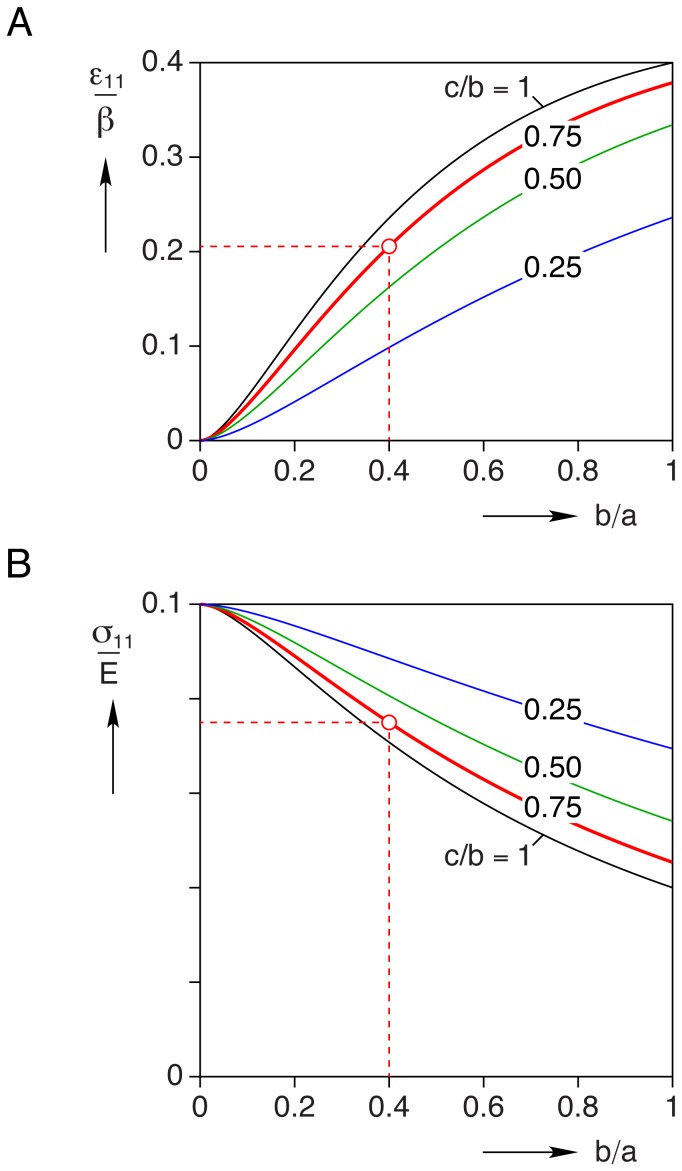
Axial strain and stress in the homogeneous inclusion. (A) strain knockdown factor (ratio of constrained strain, 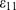, to load-free transformation strain, 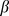) versus geometric aspect ratios of the inclusion 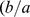, 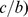, (B) normalized longitudinal stress (ratio of 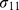 to Young’s modulus, 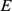).

The strain values above, when normalized by the axial (longitudinal) transformation strain 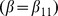, only depend on the aspect ratios of the cell (here, 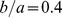, 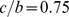). The quantity 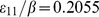, which we term the “knockdown” factor, is the ratio of *constrained* axial strain to load-free axial strain during cell contraction. For the homogeneous inclusion the knockdown is about 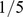. Thus, an unloaded cell that contracts by 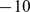 % is only able to contract to 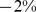 strain in a gel of the same elastic properties. Figure 2A provides curves of the knockdown as a function of the aspect ratios, 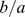 and 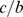, and the open circle identifies our baseline case. All curves start at the origin 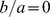, since this corresponds to the limiting case of an infinitesimally thin inclusion with no actual volume. The topmost curve for 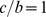 corresponds to the prolate spheroid, and the maximum value of 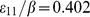 (about 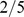) at 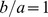 corresponds to a spherical inclusion.

For our canonical myocyte, the knockdown factor is 0.2. Note that the assumption behind this calculation is that the inclusion is a passive elastic object with 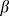 fixed. The real myocyte, however, can actively regulate its calcium signal and myofilament sensitivity in response to mechanical stress [14], [15], and hence the actual knockdown factor is likely to be less severe than the above theoretical calculation. This would require, however, that the magnitude of 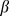 increase, thereby making the *stress* larger. One could then reinterpret 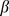 as no longer a material constant, but rather a dynamic function of mechano-chemo-transduction processes. Measuring the constrained strain of the cell and knowing the properties of the gel would, in principle, allow this function to be identified. In any event, the curves in Fig. 2A show that for a given transformation strain, the knock-down factor is less (i.e. less contraction is possible) for a slender cell (such as an atrial myocyte) than a stout cell (such as ventricular myocyte).

#### Inclusion stress & surface traction

The stress state inside the homogeneous inclusion, corresponding to eq. (44), is calculated to be
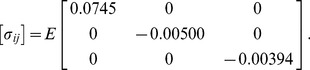
(45)


The non-zero values above are in fact normalized principal stresses, showing that the longitudinal stress is tensile 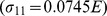 while transverse stresses 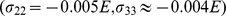 are compressive, as would be expected. While the stress state is multiaxial, it is approximately uniaxial considering the relative values 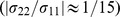. The maximum shear stress is easily calculated as 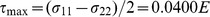. Figure 1B shows the dependence of the normalized longitudinal stress (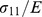) on the aspect ratios of the inclusion. The curves show that the stress in a slender cell is higher than that of a stout cell for a given transformation strain and cell stiffness. This suggests a high sensitivity of slender atrial myocytes to constraint conditions, which may explain the observation that stretch of the atria is a main contributor to atrial fibrillation and structural remodeling [16]. High blood pressure and excessive ventricular wall stress can also cause ventricular arrhythmias and fibrillation [17].

**Figure 3:**
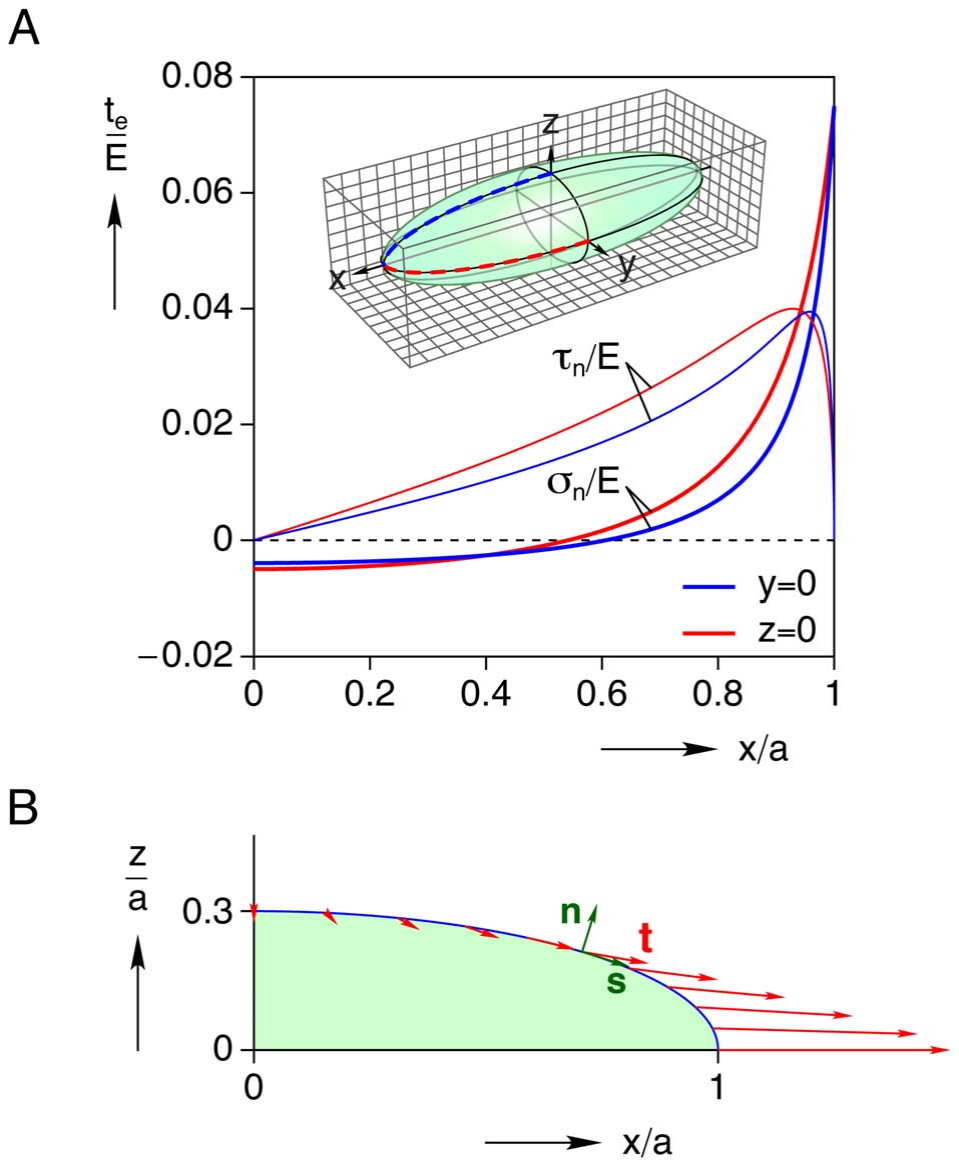
Traction distribution along the boundary of the homogeneous inclusion for the baseline case (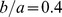, 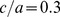). (A) normal 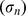 and shear 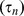 components of traction vector along contours in the planes 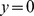 and 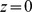, (B) scaled traction vector distribution along 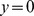 contour.l.

The surface traction distribution is also of importance, considering that various signaling molecules that reside near or on the cell membrane. While the stress state is uniform within an ellipsoidal inclusion, the traction distribution on its boundary is not. The traction vector 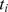 is calculated from eq. (1b), where
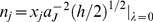
(46)is the unit outward normal to the surface in terms of 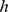 given in eq. (20). Figure 2A provides the (normalized) normal and shear traction distributions along the corresponding dashed contours in the inset. To give a sense of magnitude and direction of the traction along boundary points, Fig. 3B provides a scaled schematic of the traction vector distribution in the positive quadrant of the 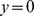 plane. The normal component of the traction is 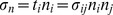, and Fig. 3A shows it changing from slight compression (over about 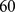 % of the length) to rapidly increasing tension until a maximum value of 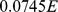 at the apex (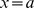). The shear component of the traction is 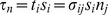, where 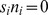, and the figure shows how it starts at zero at the waist 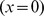, rises almost linearly across the length, but then reaches a maximum and steeply drops to zero near the apex. In each case, the variation between different contour lines is relatively minor, since the aspect ratio 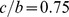 is not far from axisymmetric.

Correlating the spatial distribution of strain and stress with cellular architecture provides important insights on how mechanical load is supported by cellular structures and how mechanical stress is transduced by macromolecular complexes to affect biochemical reactions. One important finding of our analysis is that the stress state within the cell is uniform, at least from a continuum viewpoint. Hence myofilaments are expected to bear a uniform distribution of the strain and stress throughout the entire cell during contraction. The myofilament is composed of the thick myosin filament, the thin actin filament, the titin filament, and associated proteins. Upon excitation, the thick filament pulls on the thin filament to generate active contraction of the myocyte, while the titin filament provides passive elastic constraint during both stretch and contraction [18]. Titin also contains catalytic kinase domains and serves as a mechano-chemo-transducer [19]. A uniform distribution of the strain and stress across the cell suggest a uniform activation of mechano-chemo-transduction inside the cell.

Another important finding is that the traction distribution on the cell surface is highly non-uniform. This is expected to generate non-uniform strain and stress in the extracellular matrix, the cytoskeleton network, and the intercalated disk [20]. The extracellular matrix covers the cell surface and is linked to the cytoskeleton inside the cell via molecular interactions from integrin to costamere to z-disk proteins. The intercalated disk forms end-to-end attachment between adjacent cells and is linked to the cytoskeleton and myofibril via fascia adherens and cadherin complexes. Some proteins in these complexes also serve as mechano-chemo-transducers that respond to mechanical stress and activate integrin-linked kinase signaling pathways to regulate the muscle contraction and hypertrophic gene expression [21]. Our analysis show a mapping of the non-uniform traction on the cell surface (Fig. 3), suggesting that the stress is relatively low in the extracellular matrix at cell’s waist but increases sharply towards the cell’s apex, and the highest stress level exists near the intercalated disks at the apex.

#### Interior and exterior mechanical fields

**Figure 4:**
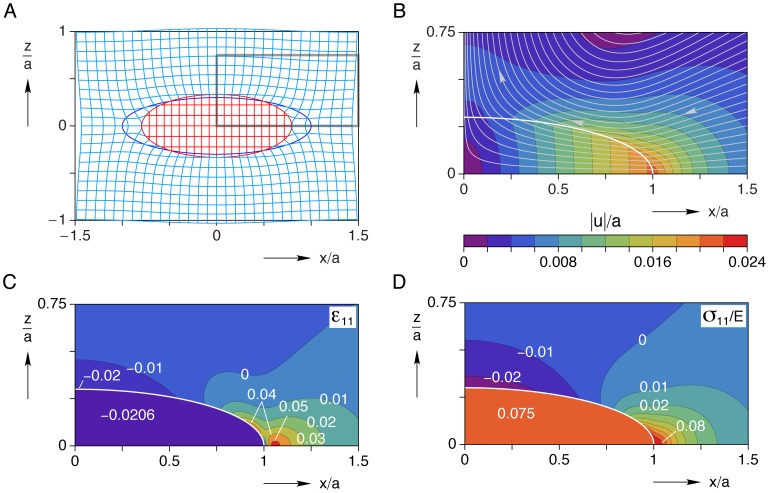
Interior and exterior mechanical fields in the 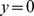 plane for the baseline case (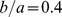, 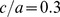). (A) deformed grid (displacements magnified 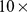), (B) magnified view of positive quadrant showing displacement streamlines and contours of normalized displacement magnitude 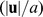, (C) longitudinal strain 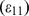 field, (D) normalized longitudinal stress 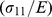 field (Young’s modulus, 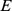).

Selected field quantities (displacements, longitudinal strain, and longitudinal stress) in the inclusion and matrix are shown in Fig. 4 in the plane 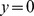. This is a symmetry plane, so 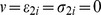 everywhere. Figure 4A shows a deformed grid where the displacements have been magnified ten-fold to clearly show the constrained inclusion which pulls on the surrounding matrix along the 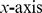 yet pushes outward on the matrix along the 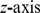. Figure 4B shows a contour plot of the magnitude of displacements 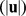, normalized by the inclusion’s half-length 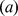 in the positive quadrant of the 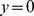 plane. Streamlines are overlaid to show the directions of displacements during contraction, again showing how the matrix is drawn inward toward the apex of the inclusion at 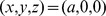 yet pushed away from the cell toward the waist at 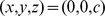. The contour plot also shows that non-zero displacements are localized in the vicinity of the cell and rapidly approach zero as 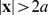, or so. Thus, this displacement map provides useful information about the expected displacements in the matrix and the spatial extent where useful displacement measurements of embedded beads can be made. Contour plots of the longitudinal strain 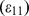 and normalized longitudinal stress 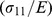 in this same region are shown in Figs. 4C and 4D, respectively. Both show ‘hot-spots’ in the matrix near the apex of the cell, and at the waist (although less severe). One can see that the active regions of stress and strain in the matrix are confined to less than one half-cell length in extent along the 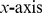 and somewhat less along the 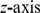.

### Inhomogeneous Inclusion Analysis

**Figure 5:**
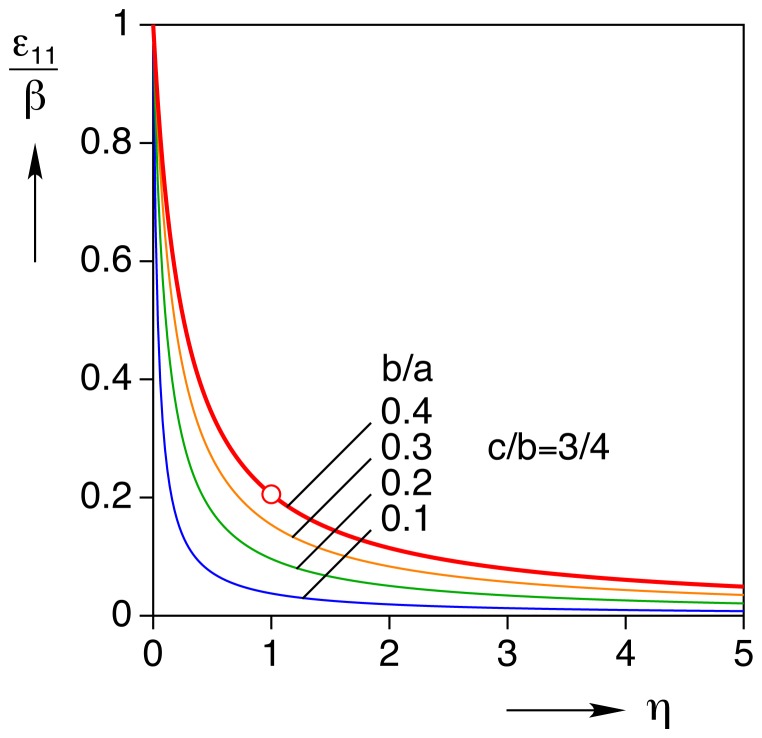
Axial strain knockdown factor for the inhomogeneous inclusion. Axial constrained strain of the inclusion 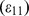 normalized by transformation strain 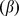 is plotted against matrix/inclusion modulus ratio 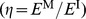 for several inclusion aspect ratios 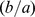 with 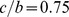 fixed.

For the case when matrix mechanical properties are different from the inclusion (the inhomogeneous inclusion) the calculated knockdown factors are provided in Fig. 5. The red curve is for the cell with the nominal dimensions given (baseline case). The other curves correspond to slender cells (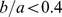, with fixed 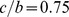). As expected, all curves decrease monotonically with the modulus ratio 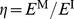, i.e. a stiffer matrix results in a smaller constrained strain magnitude. Conversely, the limiting case of 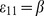 is recovered when 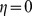, corresponding to no constraint from the matrix (load-free in solution, 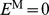).

**Figure 6:**
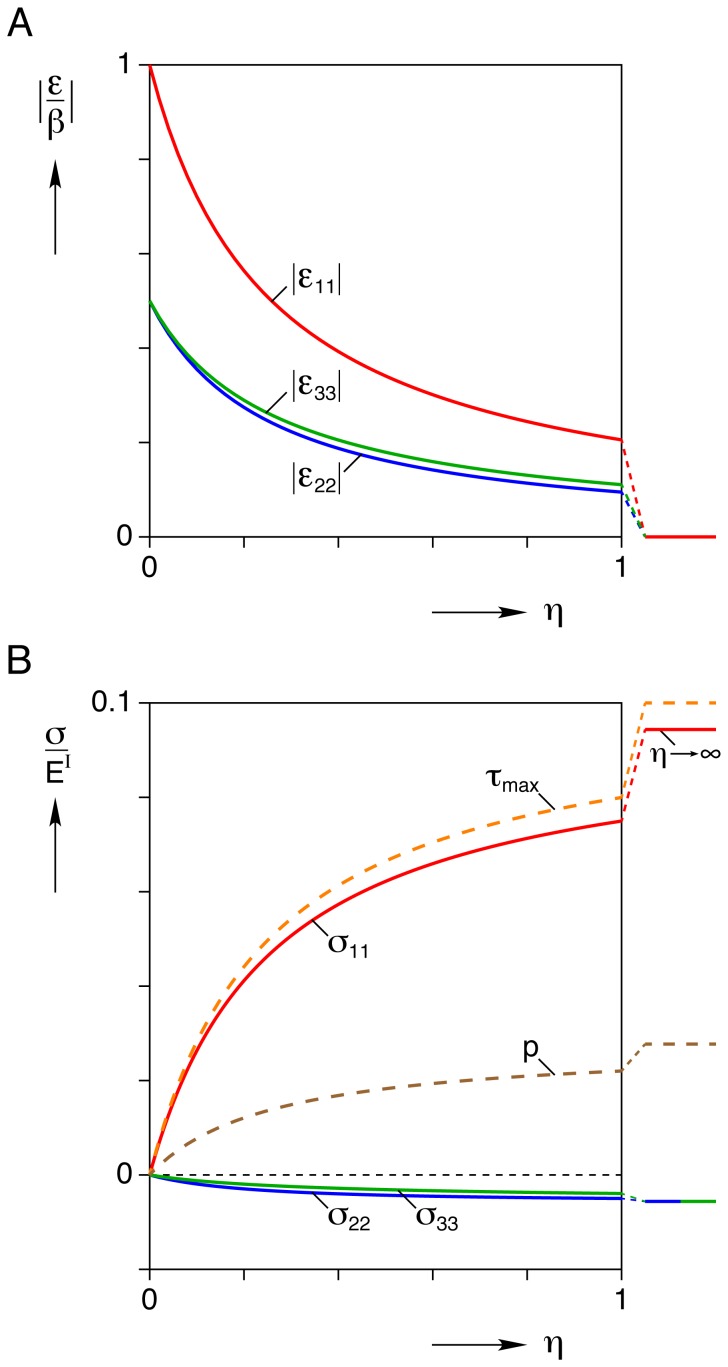
Inhomogeneous inclusion results (baseline case 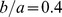, 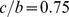) versus modulus ratio 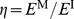. (A) strain magnitudes 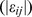 are normalized by the magnitude of transformation strain 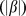, (B) stress components 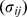, mean stress 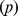, and maximum shear stress 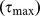, each normalized by inclusion modulus 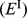.

For our baseline case, Fig. 5 shows further details of how strain components and various stress measures depend on the modulus ratio 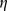. As shown in Fig. 6A, the magnitudes of all strain components decrease monotonically toward zero as the stiffness of the gel becomes large compared to that of the cell 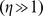. The magnitudes of the corresponding stress components (normalized by the elastic modulus of the inclusion/cell, 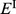), on the other hand, increase monotonically from zero at 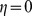 (no gel) to finite limiting values as 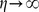 (rigid gel). The mean hydrostatic stress 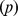 and the maximum shear stress 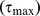 are plotted with dashed lines and these follow similar trends.

Until now, we have provided dimensionless plots in the interest of generality. Given that we have a linearly elastic solution, the strains are proportional to 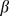 and the stresses are proportional to 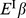, so that the provided curves can be easily scaled to obtain actual strains and stresses once the true values of 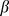 and 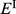 are known. For example, based on recent force-strain data acquired by longitudinal stretching of single cardiomyocytes [22], we estimate the elastic modulus of a contracting cardiomyocycte to be of the order 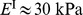. This means that the longitudinal stress of a cardiomyocyte contracting (with 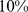 load-free contraction) in a gel of similar properties 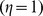 would be about 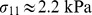, and the “blocked” longitudinal stress in the cell within a very stiff gel 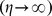 would be roughly 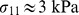.

### Energy Requirements and Mechanical Work Output

**Figure 7:**
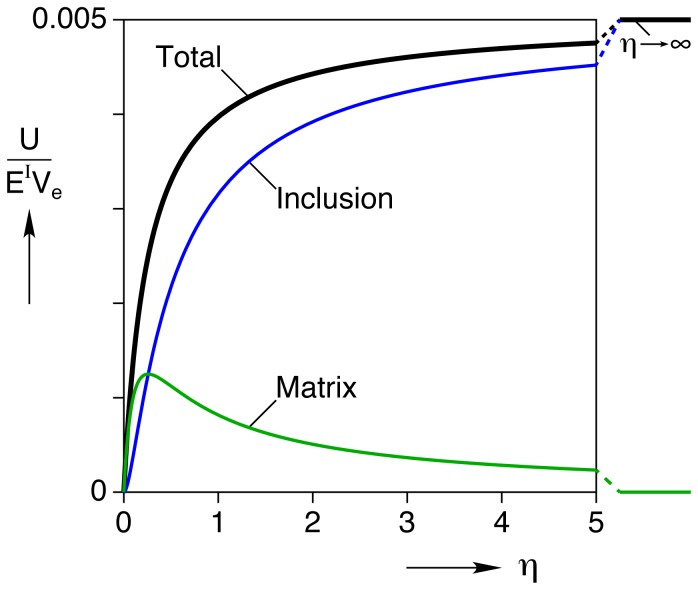
Normalized strain energy 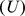 for the inhomogeneous inclusion problem versus modulus ratio 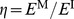. The strain energies are normalized by the inclusion modulus 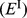 and inclusion volume 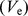 and are calculated for the baseline case (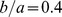, 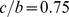).

An interesting outcome of our analysis is the ability to calculate the mechanical energy expended by the single myocyte during contraction. The total elastic strain energy by eq. (43a), using eqs. (44) and (45), is 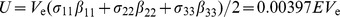 for the homogeneous inclusion. For the inhomogeneous inclusion, Fig. 7 shows the corresponding dimensionless strain energy, 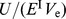, as a function of the modulus ratio 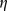, a useful result to estimate energy requirements of the cell in various constraining gels of different stiffness. The bold line shows the total strain energy, and the thin lines show the contributions in the inclusion and matrix. All curves start at zero energy when 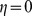. We recognize that in the absence of any external mechanical loads the cell still has certain internal energy requirements to achieve contraction (notably the “strain energy” to compress titin proteins and other internal elastic components), but here we are interested in the additional strain energy arising from *external* sources to the cell so we take this baseline energy to be zero. According to this definition, the total strain energy rises monotonically as 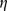 is increased and reaches a finite limiting value as 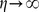. Incidentally, using 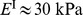 and 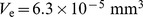, gives a strain energy of 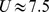 pJ for the case of the homogeneous inclusion. Most of this energy is carried inside the inclusion 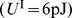, and it follows a similar trend approaching the same limiting value as the total energy. The work done by the cell on the gel is 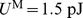. The strain energy in the matrix, however, is non-monotonic with 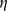, rising at first, reaching a peak value of 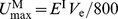, then decreasing toward zero. This is a sensible result, since in either limiting case (zero force, or zero displacement) 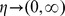 the work done on the gel is zero.

In the case of our canonical myocyte contracting in a gel of the same stiffness, the total strain energy estimated from our mechanical analysis is about 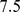 pJ per cell contraction. This value is close to the 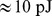 work output by a single myocyte contraction measured in the carbon fiber experiment by Bollensdorff et al. [2].

The mechanical stress states are certainly different in a myocyte in the cell-in-gel system versus the carbon fiber system, but considering the finite stores of ATP in the cell it seems reasonable to compare the two on an energetic basis. In non-biological mechanical systems, the elastic energy is often successfully used to compare structural systems with very different stress and strain states, and the energy turns out to be the unifying measure for many failure analyses. Accordingly, the energy (or more precisely, energy density) puts a given myocyte subject to various boundary conditions on a similar footing, thereby allowing a comparison of their mechanical behaviors. Also, the energy density should be less sensitive to the broad variability in myocyte shape than other measures (such as stress and strain fields). The fact that the energies are similar between the cell-in-gel model and the carbon fiber experiment seems to support this view, but direct comparison to experimental data is needed to provide more convincing proof. We note that the total energy required for myocyte contraction should include not only this mechanical energy but also the energy used to maintain metabolic homeostasis, ionic homeostasis, Ca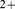 signaling and other cellular processes. Nevertheless, our analysis shows that the energy required for a single myocyte contraction is dependent on the gel stiffness, which importantly suggests that the energy expenditure/requirement should be higher for the myocyte contraction under pathological conditions with increased stiffness in myocardium, such as infarction, fibrosis, etc.

## Limitations and Perspectives

The current analysis provides an analytical solution that readily lends itself to parametric studies. As shown, it gives a useful first-order analysis of the magnitudes of constrained strains, stresses, and energies involved, which provides guidance for using cell-in-gel experiments to investigate mechano-chemo-transduction of myocytes under various mechanical loads.

As noted above, the results above were largely provided in dimensionless form to keep the results general and to investigate trends. Specific values, where needed, were cited only for illustration purposes. In particular, the value 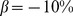 was chosen as a typical value, which seemed reasonable in light of our preliminary results on isolated healthy myocytes, as well as the results of [23] which measured end diastolic sarcomere length (SL) at 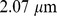 and 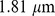 at end systole in the whole heart (about 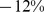 strain). However, it is well known that larger contraction strains are typically observed under preload, as when these researchers overstretched myocytes to an end-diastolic length of 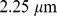 and measured an end systolic length of 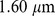
m (about 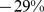 strain). Any particular value of 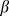 should not be viewed as a canonical value or a model limitation. As experimental results become available, the value of 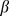 (and the aspect ratio of the cell) will be calibrated to measurements on each myocyte, and then a quantitative comparison of the model to experimental data can be performed.

As mentioned previously, our mechanical analysis treats the myocyte as an idealized elastic, yet contractile, entity rather than a real live myocyte that can actively regulate its contractile force via mechano-chemotransduction, which contributes to the Anrep effect where increased afterload enhances contractility [24]. The active regulation of myocyte contractility via mechano-chemotransduction is of particular interest for which the cell-in-gel system was designed to investigate. The mechanical analysis here serves to quantify force generation by the myocyte at a basal level (no active regulation), so we can quantitatively evaluate the enhancement of contractility above the basal level (with active regulation). Thus, this analysis builds the necessary foundation for a next study to elucidate the mechano-chemotransduction mechanisms and to investigate ‘up regulation’ of the calcium transients. The current cell-in-gel system was designed to study afterload effects on mechano-chemotransduction apart from the preload effect; therefore, the effect of preload is not yet captured. In the future, we plan to stretch the cell-in-gel system to study the preload effect, which is expected to enhance contractility, consistent with the Frank-Starling effect, and which will add another layer of complexity.

While initially motivated by the cell-in-gel experiments, we can imagine some in vivo situations where our mechanical analysis might also apply. In a normal heart where myocytes essentially beat in synchrony, the strain field is relatively uniform, albeit a transmural gradient. However, under pathological conditions asynchronous contraction (i.e. arrhythmia, fibrillation) and inhomogeneous inclusion (i.e. infarction, fibrosis) occur in the myocardium, and these can be readily simulated by the current analysis. For example, the ‘inverse’ of the current problem is that of an infarct scar in a contracting myocardium, where the local stress field around the scar would be important to know. Now the interesting region is outside the inclusion rather than inside. Our mechanical analysis still applies, but now with the sign of 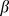 reversed. With this simple modification the stresses can still be correctly calculated. Our analyses predict that myocytes located closer to the scar region will experience a higher stress than those farther away, and the analysis quantifies the spatial dimension of this affected region. Interestingly, arrhythmogenic activities often arise from the infarct boarder zone, supporting the notion that high mechanical stress, among other factors, significantly contributes to arrhythmogenesis.

Regarding limitations, the most arguable assumption of the current analysis is that of the ellipsoidal shape of the inclusion. If one wants greater fidelity in modeling the actual shape of a particular cardiomyocycte the current analysis can be extended by the algorithm given in Rodin [13] to account for irregular polyhedra inclusions, or certainly by direct finite element simulations. Such analyses would be more complex and computationally intensive to cover a large range of parameters. Considering the significant cell-to-cell variability that exists in nature and the burgeoning parameter space necessary to capture more complex cell shapes, the benefit/cost of such alternatives is in doubt. We expect the results of a finite element analysis would show only a minor second-order difference in stress and strain fields between an ellipsoid and a more realistic shape, such as a brick-like ovoid. The existence of a (relatively) simple analytical solution for the ellipsoid was a fortuitous development that we happily exploited. For the purpose of a quick parameter study, an analytical solution is almost always preferred to a more involved numerical approach. Hence, the current analytical model sets a useful foundation for future studies as more complex features are desired.

Another extension that might be useful is to consider the case of linear viscoelastic cells and/or gels. For certain boundary value problems, of which the current analysis is one, the “Correspondence Principle of Viscoelasticity” [25] can be used when the corresponding linear elasticity solution is known. The approach only involves replacing the elastic properties by their complex counterparts to obtain the time-dependent solution. We will pursue this extension as needed, depending on the experimental results and a viscoelastic characterization of the gel and cell planned for the near future.

## Concluding Remarks

Mechanical stress is known to have significant impact on the heart function and disease development. What remains a major mystery is the cellular and molecular mechanism(s) that transduce stress to affect cardiac myocytes. As a necessary step towards understanding the mechanical stress effects, here we provide a 3-D mechanical analysis of a single myocyte beating in an elastic matrix that simulates the mechanical environment in myocardium under certain pathological conditions. The general analytic solution facilitates parametric studies of the problem and provides a quantitative mapping of the mechanical strain and stress *inside* and *outside* the myocyte. Our analyses reveal the following phenomena of particular patho-physiological importance. The fractional shortening of myocyte is dependent on the geometric dimensions of the cell and the stiffness of the surrounding matrix. A slender or softer cell has less fractional shortening. The stress state is uniform within the cell and can be approximated as uniaxial, considering the large ratio 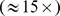 between the longitudinal and the transverse stresses. Interestingly, the surface traction is highly non-uniform. It is minimal at the waist, rises along the length, and reaches a maximum near the apex. This suggests that the stress sensing molecular complexes in the extracellular matrix and the intercalated disks should experience non-uniform distribution of the normal and the shear stress and higher stress at some ‘hot spots’, especially under certain pathological conditions (asynchronous contraction, increased stiffness due to infarction, fibrosis, etc.). Our analyses will also inform studies of the mechanotransduction mechanisms that link mechanical stress to cardiac function and remodeling in health and disease.
